# Complete genome sequence and identification of polyunsaturated fatty acid biosynthesis genes of the myxobacterium *Minicystis rosea* DSM 24000^T^

**DOI:** 10.1186/s12864-021-07955-x

**Published:** 2021-09-13

**Authors:** Shilpee Pal, Gaurav Sharma, Srikrishna Subramanian

**Affiliations:** 1 CSIR-Institute of Microbial Technology (CSIR-IMTECH), Chandigarh, India; 2 Institute of Bioinformatics and Applied Biotechnology (IBAB), Bengaluru, Karnataka India

**Keywords:** Myxobacteria, Whole-genome sequencing, Evolution, Secondary metabolites, Comparative genomics

## Abstract

**Background:**

Myxobacteria harbor numerous biosynthetic gene clusters that can produce a diverse range of secondary metabolites. *Minicystis rosea* DSM 24000^T^ is a soil-dwelling myxobacterium belonging to the suborderSorangiineae and family *Polyangiaceae* and is known to produce various secondary metabolites as well as polyunsaturated fatty acids (PUFAs). Here, we use whole-genome sequencing to explore the diversity of biosynthetic gene clusters in *M. rosea*.

**Results:**

Using PacBio sequencing technology, we assembled the 16.04 Mbp complete genome of *M. rosea* DSM 24000^T^, the largest bacterial genome sequenced to date. About 44% of its coding potential represents paralogous genes predominantly associated with signal transduction, transcriptional regulation, and protein folding. These genes are involved in various essential functions such as cellular organization, diverse niche adaptation, and bacterial cooperation, and enable social behavior like gliding motility, sporulation, and predation, typical of myxobacteria. A profusion of eukaryotic-like kinases (353) and an elevated ratio of phosphatases (8.2/1) in *M. rosea* as compared to other myxobacteria suggest gene duplication as one of the primary modes of genome expansion. About 7.7% of the genes are involved in the biosynthesis of a diverse array of secondary metabolites such as polyketides, terpenes, and bacteriocins. Phylogeny of the genes involved in PUFA biosynthesis (*pfa*) together with the conserved synteny of the complete *pfa* gene cluster suggests acquisition via horizontal gene transfer from Actinobacteria*.*

**Conclusion:**

Overall, this study describes the complete genome sequence of *M. rosea*, comparative genomic analysis to explore the putative reasons for its large genome size, and explores the secondary metabolite potential, including the biosynthesis of polyunsaturated fatty acids.

**Supplementary Information:**

The online version contains supplementary material available at 10.1186/s12864-021-07955-x.

## Background

Myxobacteria are Gram-negative, rod-shaped, soil-dwelling δ-proteobacteria taxonomically classified within the order *Myxococcales* and distributed across diverse ecological niches [1–3]. While the δ-proteobacteria are anaerobic sulfate or sulfur-reducing microbes, myxobacteria are aerobes except for the facultative anaerobe *Anaeromyxobacter* spp. and the strictly anaerobic *Pajaroellobacter* spp. [4, 5]. Unlike their close δ-proteobacteria relatives, they have large genomes (9–16 Mbp) with the exception of *Anaeromyxobacter* spp. (~ 5 Mbp), *Vulgatibacter* (4.35 Mbp), and *Pajaroellobacter* (1.82 Mbp). Apart from cellular functions, most of the functionally annotated proteins are associated with several intriguing physiological characteristics such as gliding motility, predation, fruiting body formation, biofilm formation, social behavior, etc. [6–13]. Myxobacterial vegetative cells can swarm by social and adventurous gliding in search of nutrients or for predating other microbes [3]. During starvation, myxobacterial cells (>10^5^) construct fruiting bodies which enclose myxospores that can initiate their vegetative cycle in favorable growth conditions [14].

Myxobacteria are known for their vast biosynthetic potential, as evident by the secretion of a large variety of bioactive molecules such as alkaloid, polyketide, terpene, aminocoumarin, beta-lactam, etc., produced from polyketide synthase (PKS), nonribosomal polypeptide synthetase (NRPS), and their hybrids [15, 16]. These compounds are known to have various antibiotic, antifungal and antitumor activities [17]. Most of these studied organisms belonging to *Sorangium* and *Aetherobacter* have been reported as potent producers of polyunsaturated fatty acids (PUFAs), including eicosapentaenoic acid (EPA) and docosahexaenoic acid (DHA) [18]. These *n*-*3* (omega-3) and *n-6* (omega-6) are associated with blood-pressure-lowering properties and are used for the treatment of cardiovascular diseases, diabetes, and obesity [19]. Fish oils are well-known eukaryotic sources of DHA and EPA [20] but might be contaminated with organic pollutants. Considering the huge demand for PUFA due to its health benefits, alternate PUFA synthesis via an anaerobic route integrated with a polyketide synthase (PKS) instead of the fatty acid synthase (FAS) has been explored in prokaryotes [21, 22]. This pathway for PUFA synthesis employs *pfa* gene clusters containing a total of five consecutive genes (*pfaA, pfaB, pfaC, pfaD,* and *pfaE*) in marine microorganisms such as *S. pneumatophori* SCRC-2738, *M. marina* MP-1, and *P. profundum* SS9 [22–24]. Recently, these *pfa* gene clusters have also been explored in non-marine terrestrial myxobacteria *Aetherobacter* sp. SBSr008, *Aetherobacter fasciculatus* SBSr002, and *S. cellulosum* So ce56 in producing arachidonic acid (ARA), DHA, EPA as well as linolenic acid (LA), γ-linolenic acid (GLA), stearidonic acid (SDA), and docosapentaenoic acid (DPA) [18]. Unlike marine microorganisms, myxobacterial *pfa* gene clusters include only four genes i.e., *pfa1* (homolog of *pfaD*), *pfa2* (homolog of *pfaA*), *pfa3* (homolog of *pfaC*), and a homolog of *pfaE* gene [18]. These Pfa proteins contain various domains and catalytic sites such as Pfa1 (PfaD) in *Aetherobacter* contains enoyl reductase (ER) domain, multi-functional Pfa2 (PfaA) protein contains several domains, i.e. β-ketoacyl synthase (KS), malonyl/acyltransferase (MAT/AT), acyl carrier protein (ACP), ketoreductase (KR) and PKS-like dehydratase (PS-DH) domains; and Pfa3 (PfaC) has KS, chain length factor (CLF), acyltransferase (AT), Fab-A like dehydratase (DH), pseudo-domain dehydratase (DH’) and 1-acylglycerol-3-phosphate O-acyltransferase (AGPAT) domain. In addition to these consecutive genes, another gene *pfaE* encodes for 4′-phosphopantetheinyl transferase (PPTase) [25] and is located at a separate locus in *Aetherobacter* and *S. cellulosum* So ce56 genomes. These domains are not similarly distributed in myxobacterial proteomes [18]. For example, the AT domain, seen in Pfa3 of *Aetherobacter*, is not present in *S. cellulosum* So ce56. Diversity of these domains have been reported to cause product variations from *pfa* gene clusters of terrestrial myxobacteria [18].

To characterize and explore the huge biosynthetic potential of myxobacteria, whole-genome sequencing of more strains is needed. Here we report the complete genome sequence of *M. rosea* DSM 24000^T^ and identify several biosynthetic gene clusters including one involved in the synthesis of PUFA. We also perform comparative genome analysis of *M. rosea* and other related myxobacteria to glean insights about the expansion in genome size that makes the *M. rosea* DSM 24000^T^ genome the largest bacterial genome known to date.

## Results and discussion

### Genomic properties of *M. rosea* DSM 24000^T^

**Fig. 1:**
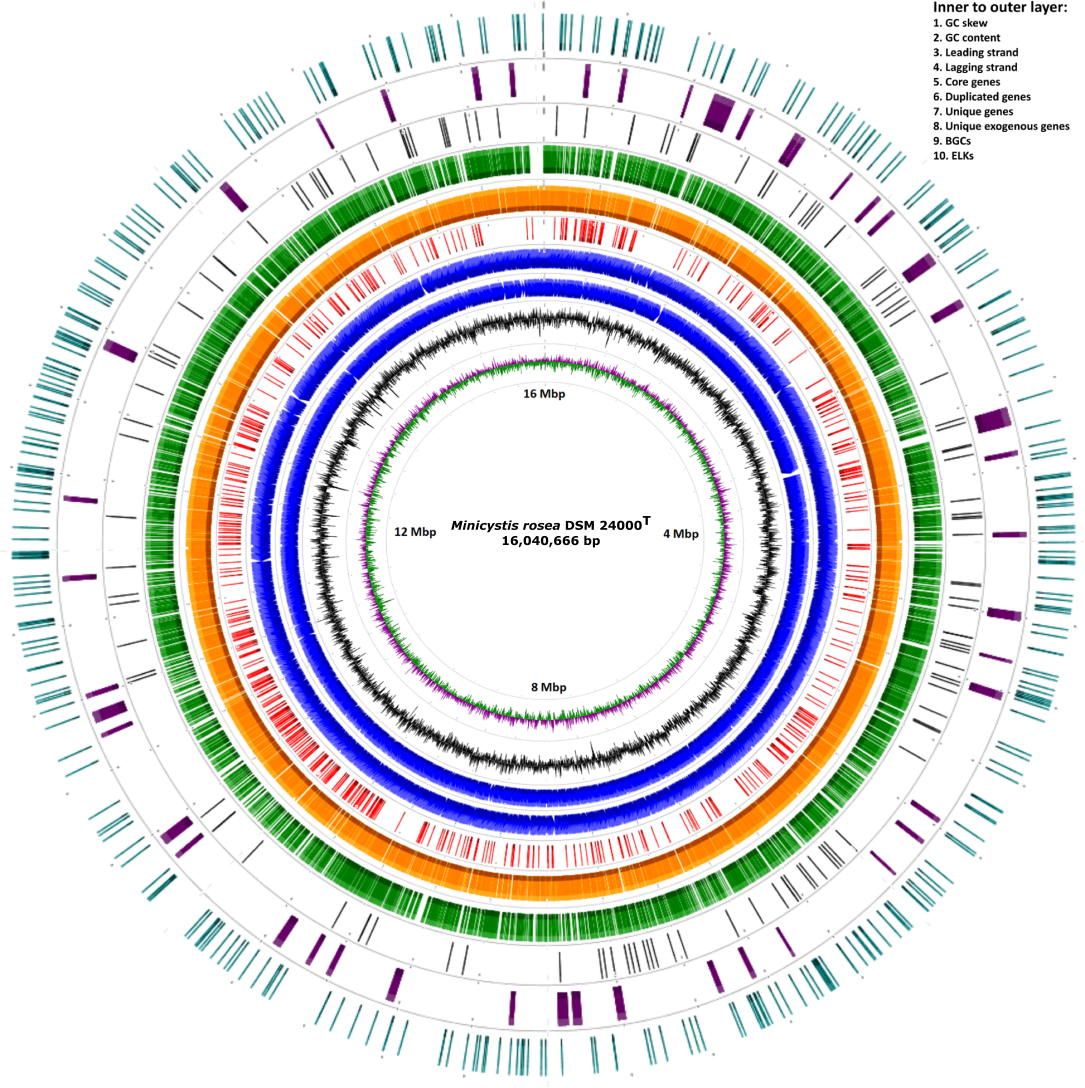
Circular representation of the genome of *M. rosea* DSM 24000^T^ showing GC skew, GC content, genes on leading and lagging strands, core genes, duplicate genes, unique genes, unique exogenous genes, secondary metabolites producing genes (BGCs), and eukaryotic-like kinase (ELK) synthesizing genes from inner to outer layers respectively

The *M. rosea* genome assembled into a complete circular chromosome of length 16,040,666 bp with 69.07% GC. It has been deposited in GenBank under the accession number CP016211.1 within the BioProject number PRJNA321464. The assembly process did not detect any plasmid sequence. This is not surprising as among the genomes of order *Myxococcales*, only one organism *M. fulvus* 124B02 has been reported to harbor a plasmid, pMF1 [26]. However, as we have used Bluepippin size selection in our sequencing, we might have missed any smaller size plasmid. RAST-based annotation has predicted 14,121 genes that consist of 14,018 protein-coding genes, 88 tRNAs, four 5S–16S-23S rRNA operons, one transfer-messenger RNA, and two non-coding RNAs (each belongs to RNase_P_RNA and SRP_RNA class) (Table 1). As of date, the genome sequence of *M. rosea* is the largest amongst kingdom bacteria (Fig. 1), and is ~ 1.26 Mbp larger than the genome of the myxobacteria *S. cellulosum* So0157–2 (14,782,125 bp), which has been previously reported as the largest prokaryotic genome [27].

**Table 1:** Assembly and annotation statistics for the complete genome sequence of *M. rosea* DSM 24000^T^

Organism name	*Minicystis rosea* DSM 24000 T
Sequencing data	PacBio P6C4 chemistry
Total reads	4,41,539
Total bases	3,48,84,02,643 bp
Average read length	7,900 bp
Average reference coverage	217X
Bio-project number	PRJNA321464
NCBI Accession number	CP016211.1
Genome size	16,040,666 bp
GC content	69.07%
Chromosome	1
CDS	14,018
Coding density	87.31%
CDS from (+) strand	6,983
CDS from (−) strand	7,035
tRNA	88
5S–16S-23S rRNA	4
tmRNA	1
ncRNA	2
Max. CDS length	22,116 bp
Mean CDS length	1,003 bp
Genes containing Pfam domains	7,844 (55.96%)
Genes with COG identified	6,275 (44.76%)
Hypothetical proteins	5,503 (39.26%)

**Fig. 2:**
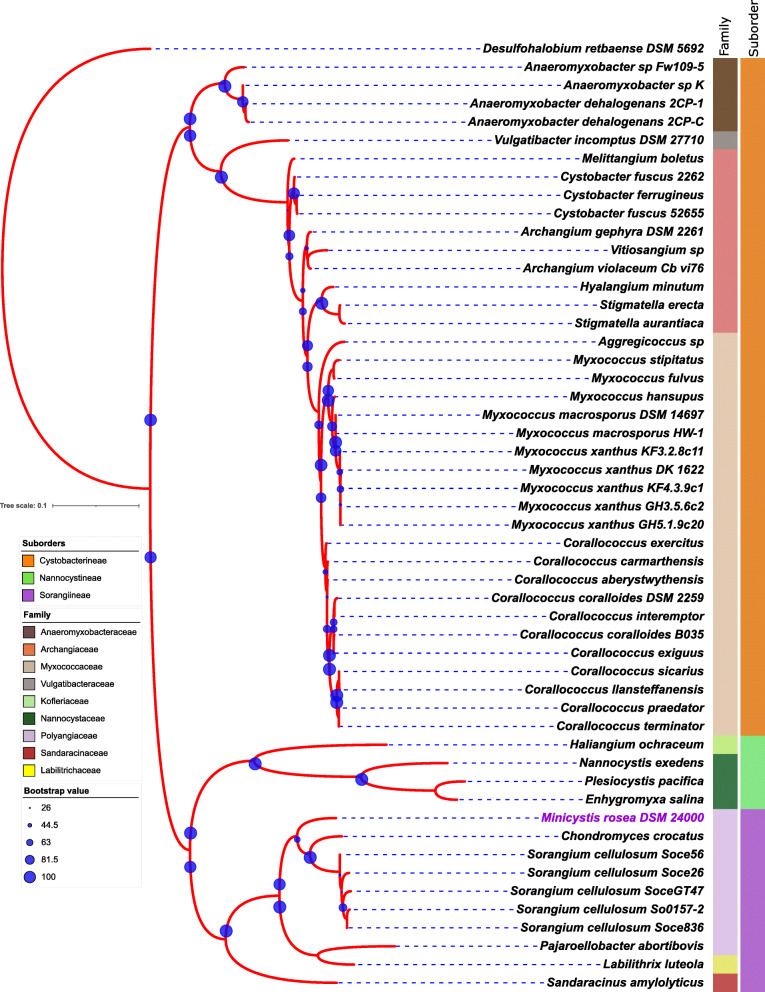
16S rRNA-based phylogenetic tree shows a close association of *M. rosea* with the members of the family *Polyangiaceae* in suborder Sorangiineae. The left and right stripes represent the suborder and family-level taxonomy (color-coded), respectively

16S rRNA-based phylogenetic tree indicates that *M. rosea* DSM 24000^T^ is a close relative of members of the family *Polyangiaceae* in suborder Sorangiineae (Fig. 2). Similar tree topology has also been observed in the marker-gene-based tree where *M. rosea* is closely clustered with selected species of the genera within the *Polyangiaceae* family (Fig. S1). Moreover, *M. rosea* also shows higher DDH and ANI values with the *Sorangium* spp. as compared to other myxobacteria (Table S1) suggesting their close relatedness.

### Analysis of genome expansion and protein function in *M. rosea* DSM 24000^T^

*M. rosea* encodes 14,018 protein-coding sequences which account for 87.50% coding density with an average gene size of 1003 bp (Table 1). A total of 6,167 (~ 44%) coding sequences have been annotated as hypothetical proteins in *M. rosea.* Our pan-genome studies with 19 other myxobacteria (having > 9 Mbp genome size) revealed vast diversity among all studied members.

#### Core genome

Our study suggested that 650 orthologous protein-coding genes are conserved and constitute  the core genome. This category includes only 5.03% of *M. rosea* proteins in contrast with its vast gene content (Table S2a). COG-based functional characterization of core proteins in *M. rosea* reveals that ‘Metabolism’ [MET] (44.14%) representation is higher than ‘Information Storage and Processing’ [ISP] (28.76%) and ‘Cellular Processes and Signaling’ [CPS] (27.70%). Most of the core proteins in *M. rosea* are involved in translation [J] (16.59%), coenzyme metabolism [H] (8.68%), lipid metabolism [I] (8.07%), energy production [C] (7%), post-translational modification [O] (6.85%), amino acid transport [E] (6.39%), transcription [K] (6.24%), cell wall biogenesis [M] (5.78%), replication [L] (5.78%), nucleotide metabolism [F] (5.33%), and signal transduction [T] (5.02%) (Fig. S2).

#### Accessory genome

**Fig. 3:**
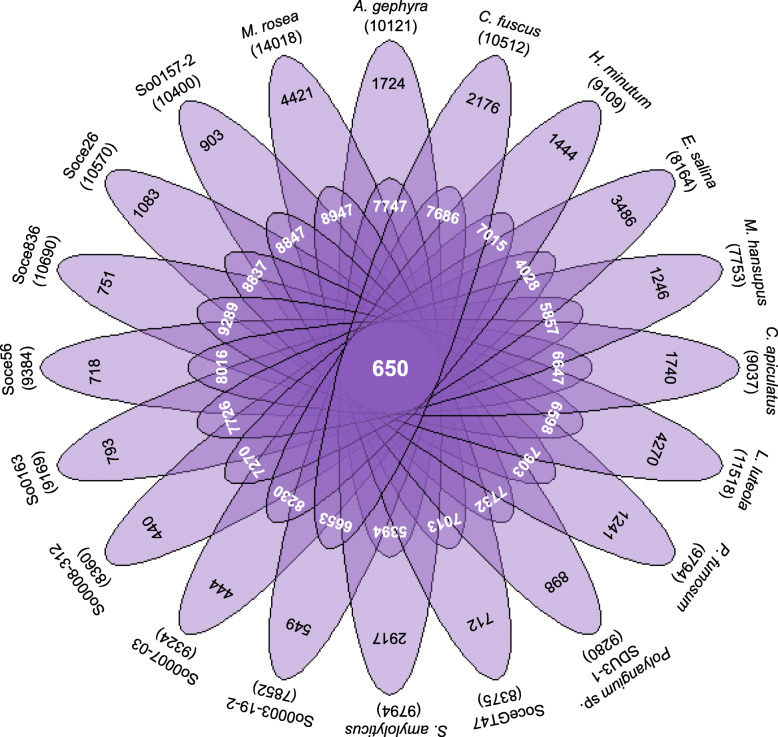
Flower plot representing the total (outermost layer), unique (second layer) (strain specific), accessory (third layer), and core proteins (center of the plot) in *M. rosea* and other 19 myxobacteria

This study identified a total of 8947 (63.83%) accessory genes in *M. rosea* (Fig. 3), which are associated with the COG category CPS in higher number (39.29%) as compared to the MET (36.86%) and ISP (16.74%) categories. Most of the accessory proteins are involved in signal transduction [T] (17.02%), transcription [K] (10.57%), cell wall biogenesis [M] (7.33%), lipid metabolism [I] (6.56%), amino acid transport [E] (5.67%), energy production [C] (5.33%), and secondary metabolites biosynthesis [Q] (5.19%) (Fig. S2).

#### Unique genome

A total of 4421 (31.54%) proteins do not display any significant identity with selected myxobacteria, which are mentioned as unique proteins in *M. rosea* (Table S2a). Among them, only 347 unique proteins have been functionally identified which are associated with the COG category CPS (34%) followed by MET (30.25%) and ISP (13.83%). Majority of unique known proteins are involved in signal transduction [T] (12.68%), transcription [K] (10.29%), cell wall biogenesis [M] (9.80%), lipid metabolism [I] (5.76%), secondary metabolites biosynthesis [Q] (5.19%), coenzyme metabolism [H] (4.61%), and post-translational modification [O] (4.32%) (Fig. S2). Among unique proteins in *M. rosea*, 125 proteins exhibit significant similarity with exogenous genetic materials, including integrated plasmids, phages, and insertion sequence (IS) elements (Table S2a). Twenty-four genomic islands (GIs) have been identified in *M. rosea* comprising a total of 6,15,248 bp (3.84%) of the genome (Table S2b). The GIs containing unique exogenous genes (Table S2b) may help facilitate horizontal gene transfer [28]*.*

#### Signal transduction

**Fig. 4:**
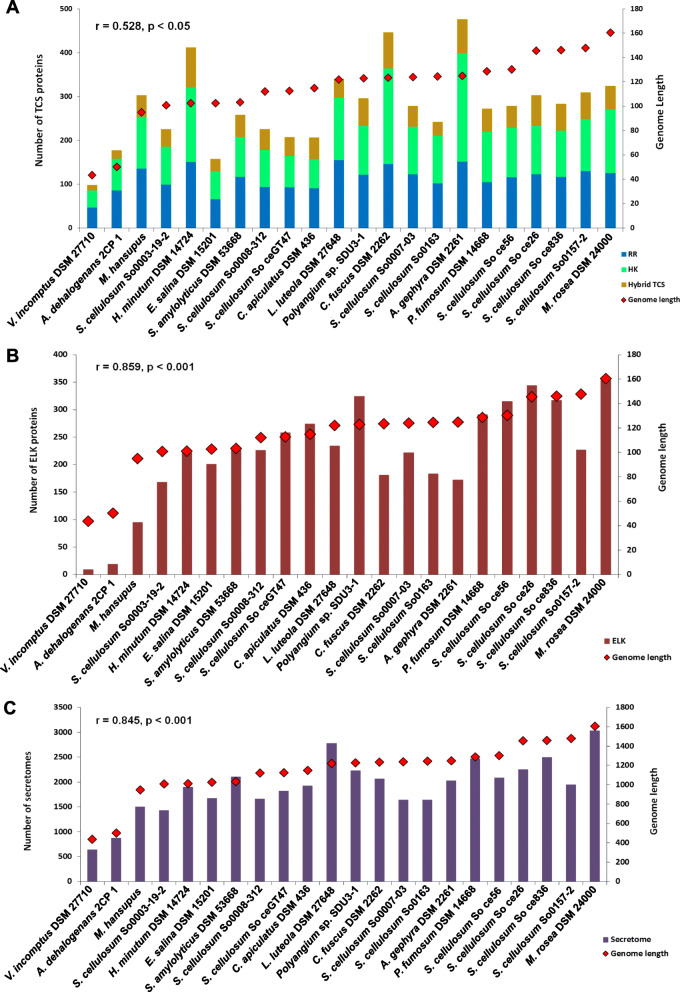
Bar plot representations of two-component system (TCS) categories [histidine kinase, response regulator, and hybrid TCS] (**A**), eukaryotic-like kinases (ELKs) (**B**), and secretome (**C**) in myxobacteria along with their genome size

Overall, our genome analysis indicates an abundance of signal transduction proteins as well as transcriptional regulators in *M. rosea.* Our analysis is supported by previous studies reporting a strong correlation between the number of bacterial transcriptional regulators and genome size [29]. Earlier, a linear relationship has been observed between the signaling proteins, including two-component system (TCS) proteins, and genome size in host-associated, as well as, environmental bacteria [30]. *M. rosea* also shows a higher number (323 proteins) of TCS proteins, which comprise 145 orphan histidine kinases (HK), 125 orphan response regulators (RR), and 53 hybrid TCS proteins as compared to *S. cellulosum* So0157–2 (309 TCS proteins) as well as other *Sorangium* spp. (Fig. 4A). However, no strong correlation (*r* = 0.531, *p* < 0.05) between the genome size and the number of TCS proteins has been found in myxobacteria, as reported previously [9]. Apart from the environmental diversity, the complex life cycle also influences the numbers of TCS proteins in the case of myxobacteria [31]. In addition to the TCS system, signal transduction mechanisms are also facilitated by serine, threonine, and tyrosine phosphorylation mediated protein kinases in prokaryotes. This protein family in myxobacteria has been reported to have strong sequence similarity with eukaryotic-like kinases (ELKs) [32]. *M. rosea* contains 353 ELKs, which is higher than *S. cellulosum* So ce56 (317) [33], as well as other myxobacteria (Fig. 4B). The number of ELKs increases with increasing genome size in bacteria [34]. A significant strong positive correlation between genome size and number of ELKs (*r* = 0.859, *p* < 0.001) is seen. In contrast to ELKs, *M. rosea* has fewer protein phosphatases (PPs) (43 genes), comprising all three major families of PPs i.e., serine/threonine PPs (PPP-family = 9 genes), metal-dependent serine/threonine PPs (PPM-family) including PP2c-type (21 genes) and SpoIIE-like PPs (5 genes), and tyrosine-specific PPs (PTP-family) including dual-specificity PTPs (5 genes), low molecular weight protein PTPs (2 genes) and PTPZ-like PTPs (1 gene). In response to the peripheral stimuli, protein kinases phosphorylate the target proteins, whereas, phosphatases deactivate them by removing the phosphate groups [35]. Thus, kinase/phosphatase ratio regulates the bacterial cell differentiation and development to quickly adapt to the persistently varying environment [36]. It has also been reported that PP2c-type PPs can compete with ELKs in bacteria [37]. However, a higher number of PP2c-type PPs has been observed in *M. rosea* (21 genes) than *A. dehalogenans* (2 genes), *M. xanthus* (4 genes), and *S. cellulosum* So ce56 (16 genes), reported as the highest PP2c-type PPs containing prokaryote [38]. Moreover, an elevated ratio of ELKs/PPs has been also observed in *M. rosea* (8.2/1), as in *A. dehalogenans* (1.7/1), *M. xanthus* (6.9/1), and *S. cellulosum* So ce56 (7.7/1) [38]. It could explain the phosphorylation events which cannot be reversed by PPs during multicellular development in myxobacteria [38]*.* We identified 90 ELK proteins as being involved in the fruiting body production in *M. rosea* by BLASTP search (length ≥ 50% and e-value ≤1e-10) against the fruiting body forming proteins of *M. xanthus* [39] and HMM-profile based searches [40]. However, crucial genes for fruiting body development (*actA*, *asgA*, *csgA*, *fruA*, and *sdeK*) identified in *M. xanthus* are absent in *M. rosea* and in *S. cellulosum* So ce56 [33]. Therefore, as suggested in earlier studies [41], it can be argued that an alternative mechanism for fruiting body development may exist in *M. rosea* [42]*.*

#### Secretome analysis

Our analysis revealed that 3035 proteins constitute the secretome in *M. rosea*, which is higher as compared to other myxobacteria (Fig. 4C). Significant positive correlation is seen between genome size and the number of secretome proteins (*r* = 0.845, *p* < 0.001). KEGG pathway analysis [43] has also revealed a higher number of proteins (104 proteins) are involved in the secretion system in *M. rosea* (KEGG pathway ID - mrm03070) as compared to *A. dehalogenans* 2CP-1 (47 proteins), *C. fuscus* DSM 2262 (60 proteins), *A. gephyra* DSM 2261^T^ (58 proteins), *M. hansupus* (53 proteins), *L. luteola* DSM 27648^T^ (35 proteins), *S. amylolyticus* DSM 53668^T^ (44 proteins), *S. cellulosum* So ce56 (67 proteins), *S. cellulosum* So0157–2 (64 proteins), and *V. incomptus* DSM 27710^T^ (27 proteins). An extensive secretion system may explain the selection of such a large number of associated genes in *M. rosea* for executing sophisticated cellular crosstalk and adaptation to diverse environments.

A variety of regulatory systems are broadly distributed across the *M. rosea* proteome, with most of them involved in transcription regulation. Free-living and soil-dwelling large-genome-containing bacteria usually acquire a complex regulatory network and a higher number of corresponding genes to survive in environments where the resources for growth are scarce but diverse [44]. Moreover, a higher number of lipid metabolism [I] associated proteins than carbohydrate metabolism [G] reveals efficient utilization of lipid as an energy source in *M. rosea* similar to that observed in *M. xanthus* [45]. Lipids have been observed in producing diverse morphological characters such as fruiting body formation in myxobacteria upon amino acid and carbon depletion [46]. Steroid biosynthesis in *M. rosea* further explores the importance of lipid bodies as signaling molecules similar to the steroid hormones in animals [47]. Thus, sophisticated intercellular communication for niche adaptation and morphogenetic variations may facilitate the retention of a huge amount of protein-coding genes in *M. rosea*.

#### Duplication events

Paralogous genes, which arise by gene duplications, comprise 44.10% genes in *M. rosea* (Table S2a). Using the same parameters to define paralogous genes, we find that well-studied members of the family *Polyangiaceae* i.e., *S. cellulosum* So ce56 and *S. cellulosum* So0157–2 contain 47.10 and 41.80% paralogous genes, respectively. Our results are in agreement with previous reports suggesting that the extensive expansion of paralogous genes account for the large genome size [48], similar to that reported in *S. cellulosum* So ce56 [33] and *S. cellulosum* So0157–2 [27]. Most of the functionally annotated paralogous proteins are involved in signal transduction [T] (21.41%), transcription [K] (12.04%), cell wall biogenesis [M] (8.08%), lipid metabolism [I] (7.30%), post-translational modification [O] (6.89%), and biosynthesis of secondary metabolites [Q] (5.47%) in *M. rosea*. Thus, the majority of gene duplications have occurred for those proteins in *M. rosea* that may help it to respond to the environmental signals and in regulatory mechanisms for niche adaptation.

#### Pfam-based functional characterization

Using HMM profile-based searches, we identified that 7446 *M. rosea* proteins were mapped to 2576 Pfam families. Comparative analysis of protein families reveals that several families such as protein kinase (360 members); histidine kinase (344 members); helix-turn-helix (315 members), TetR (139 members), transcription regulators like σ^54^ (104 members); repeats such as tetratricopeptide repeats (134 members), pentapeptide repeats (107 members), VCBS (91 members), Sel1 (16 members); phage_GPD (71 members); FGE-sulfatase (69 members); short-chain dehydrogenase (115 members); and radical SAM (70 members) are overrepresented in *M. rosea* as compared to other Sorangiineae members (*C. apiculatus*, *L. luteola*, *Polyangium* spp., *S. amylolyticus*, and *S. cellulosum*) (Table S2c). These families are associated with signaling systems, regulatory networks, protein folding, and genome packaging in *M. rosea.* Apart from these, some families such as, abhydrolase_7, aerolysin, bile_hydr_trans, creD, disintegrin, endonuclease_1, endotoxin_N, expansin_C, gluconate_2-dh3, gly_transf_sug, glyco_hydro, lectin_legB, lipase_bact_N, lipocalin, peptidase_C2, TPP_enzyme_M_2, etc. are exclusively identified in *M. rosea* (Table S2c)*.* Complex lifestyles in diverse environments might facilitate gene gain, loss, or duplication in microbes for adaptation to that niche [49]. The retention/modification of duplicated genes helps to conserve the protein functions amongst different environments [50], which could be one of the predominant causes for the large genome size in *M. rosea*.

### Biosynthetic gene clusters in *M. rosea* DSM 24000^T^ especially polyunsaturated fatty acid (PUFA) biosynthetic genes

Genome mining revealed 47 BGCs (encoded by 1081 genes) comprising 7.71% of protein-coding genes in *M. rosea*. The major fraction of biosynthetic genes encode NRPS (252 genes; 7 clusters) followed by terpene (171 genes; 9 clusters), PKS (128 genes; 4 clusters), ribosomal synthesized and post-translationally modified peptide (RiPP) (75 genes; 7 clusters), arylpolyene (75 genes; 2 clusters), lanthipeptide (73 genes; 3 clusters), RRE-containing (70 genes; 4 clusters), indole (56 genes; 3 clusters), and NRPS-PKS hybrid (30 genes; 1 cluster). Other clusters such as phosphonate (1 cluster; 38 genes), thioamitide (2 clusters; 32 genes), thiopeptide (1 cluster; 30 genes), phenazine (1 cluster; 20 genes), LAP (1 cluster; 18 genes), and siderophore (1 cluster; 13 genes) are also detected in the *M. rosea* genome. The representation of BGC genes in the *M. rosea* genome is more than the average bacterial genome (3.7%) and is similar to organisms from the genus *Streptomyces*, *Myxococcus*, *Sorangium*, and *Burkholderia* [51].

**Fig. 5:**
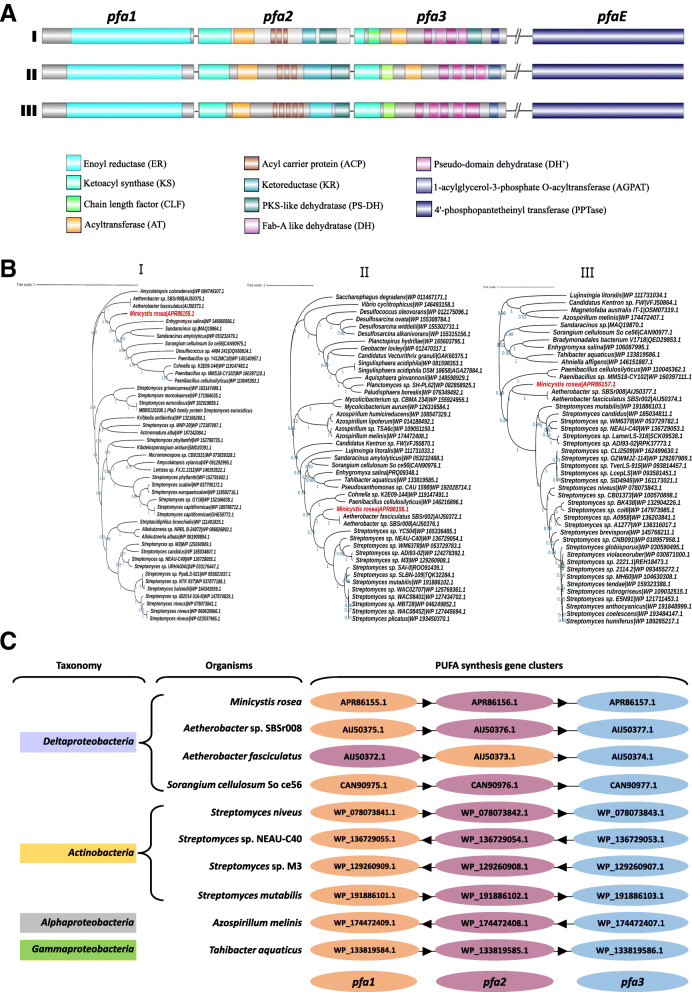
PUFA biosynthetic gene cluster organization, their phylogeny, and synteny analysis: **A** PUFA biosynthetic gene clusters and their respective domains in *M. rosea* DSM 24000^T^ (I), *Aetherobacter* sp. SBSr008 (II), and *S. cellulosum* So ce56 (III). **B** Maximum likelihood-based phylogenetic analysis shows close relatedness of the *M. rosea* DSM 24000^T^ PUFA biosynthetic proteins: Pfa1 [A7982_11504/APR86155.1] (I), Pfa2 [A7982_11505/APR86156.1] (II), and Pfa3 [A7982_11506/APR86157.1] (III) in members belonging to the genus *Aetherobacter*, *Sorangium*, *Streptomyces*, *Azospirillum*, *Tahibacter*, etc. **C** Synteny analysis of *pfa* gene cluster in *M. rosea* DSM 24000^T^ with the close relatives belonging to the genus *Aetherobacter*, *Sorangium*, *Streptomyces*, *Azospirillum*, *Tahibacter*, etc.

Further analysis of PKSs in *M. rosea* DSM 24000^T^ reveals the *pfa* gene cluster comprises four genes (*pfa1*, *pfa2*, *pfa3*, and *pfaE*) (Fig. 5) as observed in *Aetherobacter* and *Sorangium* [18]. Domain analysis of the respective proteins shows that Pfa1 (PfaD) [A7982_11504] contains a nitronate monooxygenase domain of enoyl reductase (ER) (Fig. 5AI). Sequence similarity and domain conservation of Pfa1 are seen between *M. rosea* and *Aetherobacter* spp. (Fig. 5BI). Several functional domains, i.e., β-ketoacyl synthase (KS), malonyl/acyltransferase (MAT/AT), acyl carrier protein (ACP), ketoreductase (KR), and PKS-like dehydratase (PS-DH) are positioned similarly in Pfa2 of *M. rosea* [A7982_11505] and *Aetherobacter* spp. (Fig. 5AI). The Pfa2 protein-based phylogenetic tree also reveals close relatedness between *Aetherobacter* spp. and *M. rosea* (Fig. 5BII). Pfa3 in *M. rosea* [A7982_11506] comprises several domains such as KS, chain length factor (CLF), acyltransferase (AT), Fab-A-like dehydratase (DH), pseudo-domain dehydratase (DH’), and 1-acylglycerol-3-phosphate O-acyltransferase (AGPAT) (Fig. 5AI) as detected in *Aetherobacter* spp. (Fig. 5AII). A close phylogenetic relationship is also present between the Pfa3 protein of *M. rosea* and *Aetherobacter* spp. (Fig. 5BIII). The KS domain catalyzes condensation reaction for fatty acid chain elongation [52], whereas CLF controls the fatty acid chain length [53]. MAT/AT acts as a chain extender by selecting and transferring malonic esters with the help of ACP. Other enzymes like KR, DH, and ER introduce structural diversity in the fatty acid chain. They act as tailoring enzymes that reduce intermediate keto groups, thus modifying the nascent fatty acid chain [54]. The integration of AGPAT domain into Pfa3 protein has been reported as a unique feature of the terrestrial myxobacterial PUFA synthases, which catalyzes acyl group’s transfer to generate phosphatidic acid in the chain-terminating step of PUFA synthesis [55]. Posttranslational modification of ACP occurs by the phosphopantetheinylation that converts apo-ACP to an active holo form by 4′-phosphopantetheinyl transferase (PPTase) [25]. PPTase domain has been observed in PfaE protein [A7982_13498] which is located at a separate locus of *M. rosea* proteome (Fig. 5AI) as observed in other myxobacteria like *Aetherobacter* (Fig. 5AII) and *Sorangium* (Fig. 5AIII) [18].

The acyltransferase (AT) domain is distinctly encoded by PfaB in marine γ-proteobacteria such as *S. pneumatophori* SCRC-2738, *P. profundum* SS9 [22, 23], and *M. marina* MP-1 [24]. Whereas, AT domain is integrated into the carboxy-terminus of pfa3 in *M. rosea* (Fig. 5AI) as observed in terrestrial myxobacteria *Aetherobacter* (Fig. 5AII) [18]. The domain shows 65.26% and 64.91% identities with the AT domains of pfa3 proteins in *Aetherobacter fasciculatus* and *Aetherobacter* sp. SBSr008, respectively. It plays a significant role in shaping the final PUFA products synthesized from the PUFA gene cluster. However, the AT domain is not present in pfa3 of *Sorangium* (Fig. 5AIII), which has been suggested as the reason for the inability of *Sorangium* to produce DHA and EPA [18]. Overall, homology studies suggest that the PUFA clusters in *M. rosea* and *Aetherobacter* are unique amongst myxobacteria, containing all ten enzyme domains to yield PUFAs [56] including ARA, DHA, EPA as well as LA, GLA, SDA, and DPA. The fully functional PUFA synthase in *M. rosea* enables it to produce approximately 30% of the total cellular fatty acids [57]. Overall, the phylogeny of each gene (Fig. 5B) within the PUFA cluster reveal that these PUFA genes are evolutionarily closely related to *Actinobacteria*, suggesting that *M. rosea* might have acquired these genes from *Streptomyces* species via horizontal gene transfer.

To further confirm how this cluster evolved within *M. rosea*, we also performed synteny studies based on identified homologs across close relatives. We identified that the *pfa* gene cluster in *M. rosea* along with close relatives *Aetherobacter* and *Sorangium* is completely conserved with the PUFA synthetic gene cluster in several *Streptomyces* spp., *Azospirillum melinis*, *Tahibacter aquaticus*, etc. (Fig. 5C). Conclusively, based on our phylogenetic and synteny analysis, we speculate that the *pfa* gene cluster might have been horizontally transferred to *M. rosea* and closely related myxobacteria i.e., *Aetherobacter* and *Sorangium* from Actinobacteria.

## Conclusions

Myxobacteria are well known for their large genome size and genomic content, as well as the potential to produce a wide range of secondary metabolites, including polyunsaturated fatty acids. Although there has been a huge surge in next-generation sequencing of microbes in the last three decades, however, in comparison to other soil bacteria, only a few whole-genome sequences of myxobacteria are available. In the present work, we have sequenced, assembled, and annotated a 16.04 Mbp circular genome of *M. rosea* DSM 24000^T^, the largest bacterial genome sequenced to date along with its genome characterization, and further emphasized the putative reasons for its genome expansion. Phylogenetic analysis and genome-genome distance calculation suggest *M. rosea* to be a close relative of the members of suborder Sorangiineae in the family *Polyangiaceae*. Due to its complex social behavior, diverse niche adaptation, and large genome size, *M. rosea* encodes a plethora of genes. Analysis of protein families reveals that most of the functionally identified proteins are associated with regulatory functions, protein folding, and genome packaging. Overrepresentation of protein families such as protein kinase, histidine kinase, tetR, transcription regulators like σ^54^, tetratricopeptide and pentapeptide repeats, VCBS, sel1, phage_GPD, FGE-sulfatase, short-chain dehydrogenase, and radical SAM, as well as higher numbers of secretomes and eukaryotic-like kinases in *M. rosea* as compared to other myxobacteria, are important explanations for genome expansion. Therefore, the requisite of adaptation in varied niches and complex myxobacterial multicellular behavior could be the driving forces behind genome expansion in *M. rosea*, which might be facilitated via gene-duplication followed by functional diversification of these proteins*.* A vast number of biosynthetic genes (7.71% of the coding potential) reveals the diversity of secondary metabolites production in *M. rosea.* Our study has identified the previously known functional PUFA biosynthetic gene cluster in the genome, one of the few known prokaryotic sources of DHA, EPA, LA, GLA, SDA, and DPA. Additionally, based on our phylogenetic and synteny studies, we hypothesize that this cluster might have been horizontally transferred from Actinobacteria. Our study on the genome sequencing, functional characterization, and *pfa* gene cluster analysis of *M. rosea* could further help biotechnological areas for heterologous expression of PUFAs from prokaryotes.

## Materials and methods

### Bacterial culture and isolation of genomic DNA

The actively growing plate culture of *M. rosea* was procured from Deutsche Sammlung von Mikroorganismen und Zellkulturen (DSMZ) as strain number DSM 24000^T^ (also known as strain SBNa008 or NCCB 100349). The colonies from the procured sample were subcultured on VY/2 agar (DSMZ Medium 9) plates. These actively growing subculture plates were used to isolate whole genomic DNA using Zymogen Research Bacterial/fungal DNA isolation kit and Phenol-Chloroform-Isoamyl alcohol (PCI) methods. The quantity and quality of the extracted DNA were confirmed by gel electrophoresis and Nanodrop and supported by Qubit quantification.

### Genome sequencing and assembly of *M. rosea* DSM 24000^T^

Isolated high-quality DNA was used for whole-genome sequencing (WGS) on a Pacific Biosciences RSII instrument available at the McGill University and Genome Quebec Innovation Center, Montreal (Quebec), Canada. SMRTbell long library was created with 10 mg whole genomic DNA using a 20-kb template preparation method using Procedure and Checklist-20 kb Template Preparation using BluePippin™ Size Selection (https://www.pacb.com/wp-content/uploads/Procedure-Checklist-Preparing-gDNA-Libraries-Using-the-SMRTbell-Express-Template-Preparation-Kit-2.0.pdf; last accessed: 3 Sep 2021). Later the library was loaded onto three single molecules real-time (SMRT) cells and sequenced using P6 polymerase and C4 chemistry (P6C4) with 180-min movie time. PacBio sequencing generated 4,41,539 raw reads (3,48,84,02,643 bp) with an average read length of 7900 bp. The Hierarchical Genome Assembly Process (HGAP) Pipeline v. SMRT v2.3.0 and consensus polishing with Quiver [58] were used to generate de novo assembly using default parameters. Gene prediction and functional annotation were performed by Rapid Annotation using Subsystem Technology (RAST) [59], whereas rRNA and tRNA genes were predicted using RNAmmer 1.2 [60] and tRNAscan-SE-1.23 [61]. RNAz 2.0 tool [62] was used to identify structured non-coding RNA (*P* > 0.85). A circular plot for *M. rosea* DSM 24000^T^ genome was drawn using BRIG (v 0.95-dev.0004) [63].

### Phylogenetic analysis and estimation of DNA-DNA hybridization and average nucleotide identity

The 16S rRNA sequences reported for the members of all three myxobacteria suborders i.e., Cystobacterineae, Nannocystineae, and Sorangiineae were retrieved from the NCBI database. 16S rRNA sequences from all myxobacteria and an outgroup *D. retbaense* DSM 5692 were aligned using ClustalW [64]. The alignment was used to generate a phylogenetic tree using the GTR-GAMMA model [bootstrap: 100] of maximum likelihood (ML) method in the RAxML (v8) tool [65] and visualized by iTOL [66]. We also performed phylogenetic analysis of myxobacteria using 40 universal single-copy genes (*gtp1, pheS, argS, rpsL, rpsG, rpsB, rplK, rplA, rplC, rplD, rplB, rplY, rpsC, rplN, rplE, rpsH, rplF, rpsE, rpsM, rpsK, rplM, rpsI, hisS, serS, rpsO, rpsS, rpsQ, rplP, rplO, cysS, rplR, leuS, rpsD, valS, tsaD, rpoB, rpoA, secY, ffh,* and *ftsY*) which were identified as marker genes (MGs) using fetchMGs tool (http://motu-tool.org/fetchMG.html) [67]. Nucleotide sequences of these marker genes were retrieved from each genome, aligned using ClustalW, and further concatenated. The tree was generated using the GTR-GAMMA model of the ML method [bootstrap: 100] in RAxML (v8) tool and visualized by iTOL.

In silico DNA-DNA hybridization (DDH) and Average Nucleotide Identity (ANI) were calculated between *M. rosea* DSM 24000^T^ and other 21 selected members (all representative genomes from suborder Sorangiineae and a few representative genomes from other families in order *Myxoccales*) using Genome-to-Genome Distance Calculator (GGDC) server [68] and ANI Calculator [69] respectively.

### Working data, functional characterization, and estimation of orthologous genes

As two [*Vulgatibacter incomptus*, *Pajaroellobacter abortibovis* EBA] out of 21 selected genomes have relatively smaller genome size, 19 myxobacterial representatives from three suborders of the order *Myxococcales*, i.e. Sorangiineae (*C. apiculatus* DSM 436, *P. fumosum*, *Polyangium* sp. SDU3–1, *S. cellulosum* So ce26, *S. cellulosum* So ce56, *S. cellulosum* So ce836, *S. cellulosum* So ceGT47, *S. cellulosum* So0003–19-2, *S. cellulosum* So0007–03, *S. cellulosum* So0008–312, *S. cellulosum* So0157–2, *S. cellulosum* So0163, *Labilithrix luteola* DSM 27648^T^, *S. amylolyticus* DSM 53668^T^) [33], Cystobacterineae(*A. gephyra* DSM 2261^T^, *C. fuscus* DSM 52655, *H. minutum* DSM 14724^T^, *Myxococcus hansupus*), and Nannocystineae (*E. salina* DSM 1520) [9, 70–72] were selected to perform pangenome analysis via identifying homologous and orthologous proteins using Proteinortho (v6) (https://www.bioinf.uni-leipzig.de/Software/proteinortho/) [73]. Paralogous proteins in *M. rosea* were identified by all-against-all BLAST analysis (identity ≥30% and e-value ≤1e-10) of proteomes in *M. rosea* DSM 24000^T^ using NCBI Blast+ 2.10.1 package [74]. Exogenous genetic materials in *M. rosea* DSM 24000^T^ were identified by performing BLASTP (e-value ≤1e-30) against the dataset of plasmids, phages, and insertion sequence (IS) elements retrieved from the ACLAME database (http://aclame.ulb.ac.be/). Genomic islands in the *M. rosea* genome were identified using IslandViewer 4 [75].

### Protein domains and functional analysis

Functional family and domains in all selected members of Sorangiineae were identified by scanning the Pfam-A database (v32.0) [76] using the hmmscan program (e-value ≤1e-5) of HMMER (http://hmmer.janelia.org/) [77]. Representative domains of two-component system (TCS) such as, HisKA (PF00512), Hpt (PF01627), HATPase_c (PF02518), His_kinase (PF06580), HWE_HK (PF07536), HisKA_2 (PF07568), HisKA_3 (PF07730), HATPase_c_2 (PF13581) and Response_reg (PF00072) were identified. Eukaryotic like kinases (Elks): Pkinase (PF00069), Pkinase_C (PF00433) and Pkinase_Tyr (PF07714); and protein phosphatases (PPs): PP2C_2 (PF13672, COG0631), SpoIIE (PF07228, COG2208), PPPs (PF00149, COG0639) DSPc (PF00782, COG2365) and LMWPc (PF01451, COG0394, COG2453), and PTPZ (COG4464) were explored. Functional categorization of *M. rosea* proteins was performed by estimating their Clusters of Orthologous Groups (COGs) [78] using the NCBI COG database [79]. The aforementioned gene clusters were grouped into various COG categories such as ‘Cellular processes and Signaling’ [CPS], ‘Information Storage and Processing’ [ISP], ‘Metabolism’ [MET], and ‘Poorly Characterized’ [PC] [80]. SignalP (v5.0) [81], PRED-TAT [http://www.compgen.org/tools/PREDTAT] and PRED-LIPO [http://www.compgen.org/tools/PRED-LIPO] were used to identify the secretome via signal peptide detection. Screened secretory protein sequences were used as queries on the TMHMM server, and protein sequences with 0–2 transmembrane domains were considered as final secretomes [82].

### Estimation of biosynthetic gene clusters in *M. rosea* DSM 24000^T^

Prediction of BGCs in *M. rosea* was performed using the antiSMASH tool (v5.0) (https://antismash.secondarymetabolites.org) [83] and the identified BGCs were further processed using the BiG-SCAPE program (https://git.wageningenur.nl/medema-group/BiGSCAPE) [84]. Among the estimated BGCs, PUFA producing gene cluster was identified by considering the PUFA biosynthetic genes in *Aetherobacter* sp. SBSr008 (gene accession no. - AIJ50375.1, AIJ50376.1, and AIJ50377.1), *A. fasciculatus* SBSr002 (gene accession no. - AIJ50372.1, AIJ50373.1, and AIJ50374.1), and *S. cellulosum* So ce56 (gene accession no. - CAN90975.1, CAN90976.1, CAN90977.1, and CAN95221.1) [18]. BLAST searches were performed for each of the Pfa1, Pfa2, and Pfa3 protein sequences of *M. rosea* DSM 24000^T^, and were further considered for phylogenetic analysis using the WAG (G + I + F) model of the Maximum Likelihood method in MEGA X [85]. The trees were visualized using iTOL.

## Supplementary Information

**Additional file 1: Fig. S1.** Single-copy genes-based phylogenetic tree of myxobacteria. Branch color and leaf stripes represent the suborder and family-level taxonomy (color-coded), respectively.

**Additional file 2: Fig. S2.** COG functional categorization of the Total, Accessory, Core, and Unique proteins in *M. rosea*. CPS = Cellular Processes and Signaling, ISP = Information Storage and Processing, MET = Metabolism, and PC = Poorly characterized.

**Additional file 3: Table S1.** DDH and ANI values between *M. rosea* and selected myxobacteria. Color intensity changes from green to orange corresponding with higher to lower values, respectively.

**Additional file 4: Table S2.** a) Distribution of core, unique, duplicate, ELK, BGC genes in *M. rosea.* Unique exogenous genes in Column E have been shaded using orange color.; b) Identified genomic islands in *M. rosea* genome; and c) Comparative distribution of Pfam families among the selected myxobacterial genomes.

## Data Availability

The complete genome sequence of *Minicystis rosea* DSM 24000^T^ and its annotations are deposited at DDBJ/ENA/GenBank under accession number CP016211.1.

## Abbreviations

CPS: Cellular Processes and Signaling
MET: Metabolism
ISP: Information Storage and Processing
TCS: Two-component system
HK: Histidine kinases
RR: Response regulators
ELKs: Eukaryotic-like kinases
PPs: Protein phosphatases
BGC: Biosynthetic gene cluster
PKS: Polyketide synthase
NRPS: Nonribosomal polypeptide synthetase
RiPP: Ribosomal synthesized and post-translationally modified peptide
PUFA: Polyunsaturated fatty acid
KS: β-ketoacyl synthase
MAT/AT: Malonyl/acyltransferase
ACP: Acyl carrier protein
KR: Ketoreductase
PS-DH: PKS-like dehydratase
CLF: Chain length factor
AT: Acyltransferase
DH: Fab-A-like dehydratase
DH’: Pseudo-domain dehydratase
AGPAT: 1-acylglycerol-3-phosphate O-acyltransferase
PPTase: 4′-phosphopantetheinyl transferase
