# Supplementary figures and images for: Complete genome sequence and identification of polyunsaturated fatty acid biosynthesis genes of the myxobacterium Minicystis rosea DSM 24000T
Source: BMC Genomics. 2021 Sep 13;22:655. doi: 10.1186/s12864-021-07955-x (PMC8436480; doi:10.1186/s12864-021-07955-x)

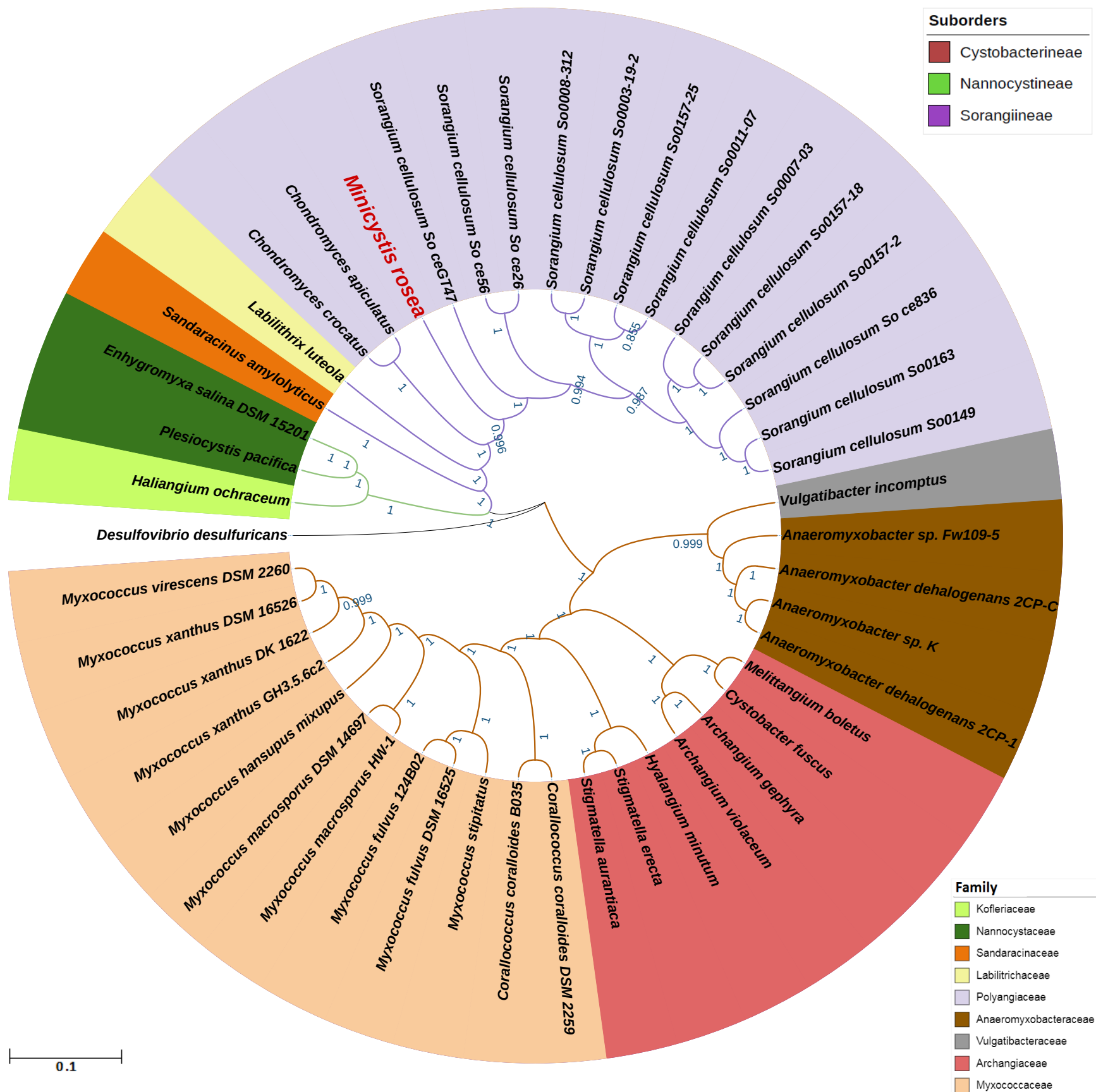

Supplement: Supplementary file 1 — Additional file 1: Fig. S1. Single-copy genes-based phylogenetic tree of myxobacteria. Branch color and leaf stripes represent the suborder and family-level taxonomy (color-coded), respectively. [file 12864_2021_7955_MOESM1_ESM.pdf]

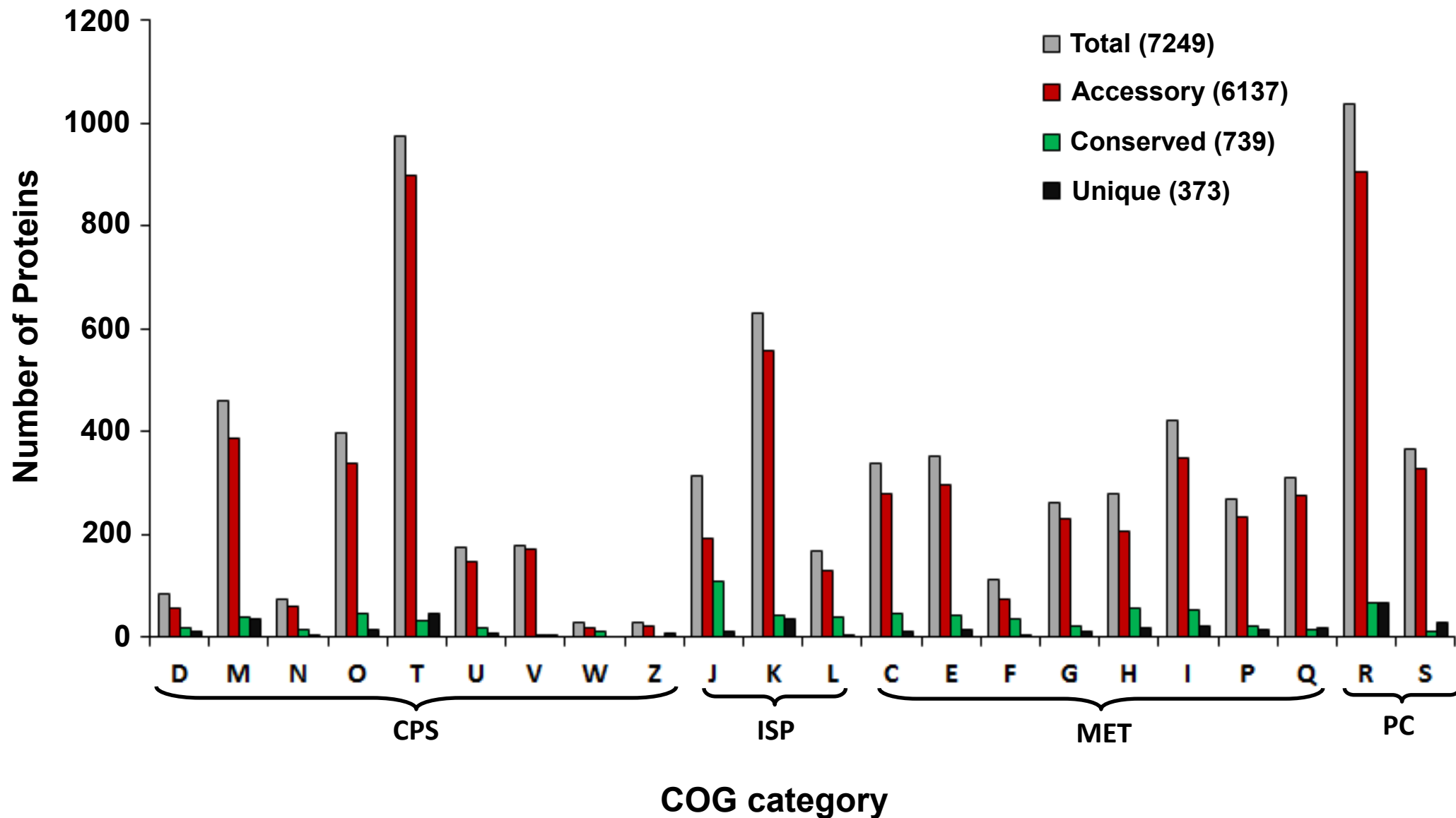

Supplement: Supplementary file 2 — Additional file 2: Fig. S2. COG functional categorization of the Total, Accessory, Core, and Unique proteins in M. rosea. CPS = Cellular Processes and Signaling, ISP = Information Storage and Processing, MET = Metabolism, and PC = Poorly characterized. [file 12864_2021_7955_MOESM2_ESM.pdf]
