# Supplementary material for: Complete genome sequence and identification of polyunsaturated fatty acid biosynthesis genes of the myxobacterium Minicystis rosea DSM 24000T
Source: BMC Genomics. 2021 Sep 13;22:655. doi: 10.1186/s12864-021-07955-x (PMC8436480; doi:10.1186/s12864-021-07955-x)
Supplement: Supplementary file 3 — Additional file 3: Table S1. DDH and ANI values between M. rosea and selected myxobacteria. Color intensity changes from green to orange corresponding with higher to lower values, respectively. [file 12864_2021_7955_MOESM3_ESM.pdf]

Supplementary Table S1: DDH and ANI values between *M. rosea* and selected myxobacteria. Color intensity changes from green to orange corresponding with higher to lower values, respectively.

Query	Sub order	Family	Members	Genome size (Mbp)	DDH	ANI value (%)
***Minicystis roseae* DSM 2400**	*Sorangiineae*	*Labilitrichaceae*	*Labilithrix luteola* DSM 27648	12.19	19.1	70.26
		Polyangiaceae	*Chondromyces apiculatus* DSM 436	11.49	19.8	72.93
			Polyangium fumosum DSM 14668	12.88	20.1	73.42
			Polyangium sp. SDU3-1	12.28	19.7	73.6
			Sorangium cellulosum So ce26	14.56	20.7	74.34
			Sorangium cellulosum So ce56	13.03	20.9	74.64
			Sorangium cellulosum So ce836	14.59	21.1	74.73
			Sorangium cellulosum So ceGT47	11.26	20.9	75
			Sorangium cellulosum So0003-19-2	10.09	21.4	75
			Sorangium cellulosum So0007-03	12.39	21.4	74.97
			Sorangium cellulosum So0008-312	11.21	20.8	75
			Sorangium cellulosum So0157-2	14.78	21	75
			Sorangium cellulosum So0163	12.46	21.2	74.86
		Sandaracinaceae	*Sandaracinus amylolyticus* DSM 53668	10.33	18.3	70.05
	Cystobacterineae	*Anaeromyxobacteraceae*	*Anaeromyxobacter dehalogenans* 2CP-1	5.03	18.8	69.52
		Archangiaceae	*Archangium gephyra* DSM 2261	12.49	18.4	68.51
			Cystobacter fuscus DSM 52655	12.35	19.1	68.72
			Hyalangium minutum DSM 14724	10.12	18.8	66.39
		Myxococcaceae	*Myxococcus hansupus* mixupus	9.49	18.5	68.19
		Vulgatibacteraceae	*Vulgatibacter incomptus*	4.35	18.1	68.56
	Nannocystineae	*Nannocystaceae*	*Enhygromyxa salina* DSM 15201	10.26	17.3	67.32

Supplementary Table S2: Excel file has been provided.
